# Costimulatory and coinhibitory molecules of B7-CD28 family in cardiovascular atherosclerosis: A review

**DOI:** 10.1097/MD.0000000000031667

**Published:** 2022-11-11

**Authors:** Mao Yang, Simeng Tian, Zhoujun Lin, Zhenkun Fu, Chenggang Li

**Affiliations:** a Department of Cardiology, Electrophysiological Center of Cardiology, The Fourth Affiliated Hospital of Harbin Medical University, Harbin, China; b Basic Medicine College, Harbin Medical University, Harbin, China; c State Key Laboratory of Medicinal Chemical Biology and College of Pharmacy, Nankai University, Tianjin, China; d Department of Immunology, Wu Lien-Teh Institute, Heilongjiang Provincial Key Laboratory for Infection and Immunity, Harbin Medical University, Heilongjiang Academy of Medical Science, Harbin, China.

**Keywords:** atherosclerosis, B7, CD28, co, co, inhibitory molecules, stimulatory molecules

## Abstract

Accumulating evidence supports the active involvement of vascular inflammation in atherosclerosis pathogenesis. Vascular inflammatory events within atherosclerotic plaques are predominated by innate antigen-presenting cells (APCs), including dendritic cells, macrophages, and adaptive immune cells such as T lymphocytes. The interaction between APCs and T cells is essential for the initiation and progression of vascular inflammation during atherosclerosis formation. B7-CD28 family members that provide either costimulatory or coinhibitory signals to T cells are important mediators of the cross-talk between APCs and T cells. The balance of different functional members of the B7-CD28 family shapes T cell responses during inflammation. Recent studies from both mouse and preclinical models have shown that targeting costimulatory molecules on APCs and T cells may be effective in treating vascular inflammatory diseases, especially atherosclerosis. In this review, we summarize recent advances in understanding how APC and T cells are involved in the pathogenesis of atherosclerosis by focusing on B7-CD28 family members and provide insight into the immunotherapeutic potential of targeting B7-CD28 family members in atherosclerosis.

## 1. Introduction

Atherosclerosis, narrowing of the arteries due to plaque buildup, is a dominant cause of many cardiovascular diseases, including myocardial infarction, heart failure, and stroke. Risk factors for atherosclerosis include high cholesterol levels, high blood pressure, diabetes, smoking, obesity, and family history; however, the exact cause of atherosclerosis is still an issue that needs to be fully understood. Atherosclerosis is now recognized as a chronic inflammatory disease in the walls of arteries, and both the innate and adaptive immune systems participate in its pathogenesis.^[1–5]^ The models of low-density lipoprotein receptor–deficient mice (Ldlr^−^/^−^) and apolipoprotein E–deficient mice (ApoE^−^/^−^) have established the vital role of T cells in the atherosclerotic process.^[6–8]^ In the early stage of atherosclerosis, leukocytes (especially T cells) and monocytes adhere to the injured vascular bed lining and transmigrate into the subendothelial space. In the atherosclerotic process, monocytes differentiate into macrophages, engulf trapped lipoprotein particles, and subsequently transform into foam cells. Chemokines from these macrophages can further increase the recruitment of monocytes, neutrophils, and lymphocytes from circulation. Foam cells can undergo apoptosis as a result of excessive lipid clearance. Failure of efferocytosis in these cells causes necrosis and forms the necrotic core of atherosclerosis.^[9,10]^ The frequency of vascular dendritic cells (DCs) in arteries increases over the course of the disease. DCs can also take up lipids and contribute to foam cell formation.^[11,12]^ Both monocyte-derived macrophages and DCs are classical antigen-presenting cells (APCs) that help activate and shape T cell responses in plaques. Activated T cells secrete inflammatory cues, such as cytokines, which enhance APC survival and effector functions. The cross-talk between APCs and T cells is crucial for the progression of vascular inflammation. The B7-CD28 costimulatory molecular family is one of the most important participants in APC-T cell interactions and has been implicated in many immune-related diseases for decades. Several recent studies have highlighted the important role of B7-CD28 family members in atherosclerosis and suggested the therapeutic potential of targeting this pathway. In this review, we first briefly summarize the functions of costimulatory and coinhibitory B7-CD28 molecules and their roles in the interaction between APCs and T cells. We then discuss the immunological mechanism of atherosclerosis formation and review the important evidence of the involvement of the B7-CD28 family in blood vessel atherosclerosis. The therapeutic potential of interfering with costimulatory or initiating coinhibitory signals by modifying B7-CD28 family members in atherosclerosis was evaluated at the end of this review.

## 2. Costimulatory and coinhibitory members on APCs and T cells

For naïve T cells to be activated, at least two signals are required. the first signal is provided by the interaction between MHC molecules expressed by APC and T cell receptors expressed on the surface of T cells; the second signal, called costimulation, is also fueled by APCs.^[13,14]^ The absence of a costimulatory signal may cause anergy or apoptosis in T cells. Targeting the costimulatory pathway is viewed as an important therapeutic strategy in many autoimmune diseases and inflammatory conditions, in which T cells are actively involved in the pathogenesis of these diseases.

There are several superfamilies of costimulators according to their different structures and functions, such as the B7-CD28 superfamily, immunoglobulin super-family, and tumor necrosis factors and their receptors superfamily.^[15,16]^ Among these families of molecules, B7-CD28 family members have been the most studied and are the most important elements in shaping T cell responses.^[17,18]^ B7 family molecules, which are expressed mainly on APCs, bind to CD28 family receptors on T cells. Notably, members of the B7-CD28 family are not always costimulators, some of which may inhibit T cell responses, a process called coinhibition (Table 1).

**Table 1:** Costimulatory and coinhibitory molecules of B7-CD28 family on T cells and antigen presenting cells in atherosclerosis.

Types	Receptors on T cells	Ligands on APCs	Potential effects on atherosclerosis (references no.)
Costimulation	CD28	B7.1(CD80)/B7.2(CD86)	^[96,100–103]^
	ICOS(CD278)	ICOSL(CD275,B7-H2)	^[105,108–112]^
	TLT-2(TREML2)	B7-H3	Unclear
	NKp30	B7-H6(NCR3LG1)	Unclear
	CD28H	B7-H5(VISTA, SISP1,VSIR)	Unclear
	CD83	TLR4/MD-2 complex	^[104,114–117]^
	Unknown	B7-H7(HHLA2)	Unclear
Coinhibition	CTLA-4(CD152)	B7.1(CD80)/B7.2(CD86)	^[54,107,119–121]^
	PD-1(CD279)	PD-L1(CD274,B7-H1)/PD-L2(CD273,B7-DC)	^[118,123–132]^
	Unknown	B7-H3	Unclear
	Unknown	B7-H4(B7S1)	Unclear
	BTLA(CD272)	HVEM/CD160	^[136,138]^
	Unknown	B7-H7(HHLA2)	Unclear

APC = antigen-presenting cell.

### 2.1. The role of costimulatory molecules of b7-CD28 family on interaction between T cells and APCs

B7.1/B7.2 (also known as CD80 and CD86) are the best-studied costimulatory molecules within the B7 family. While B7.1 is rapidly upregulated in APCs by stimulation with LPS, B7.2 is mainly expressed by APCs in resting states. By interacting with their receptors CD28, B7.1/B7.2 can elicit T cells to produce IL-2, which acts on T cells to induce T cell proliferation. In addition, signals through CD28 confer vital survival signals to activated T cells via the Bcl-xl pathway.^[18–20]^ Another costimulatory receptor, CD83, regulates B cell activation, germinal center responses, and CD4 + T cell development.^[21–23]^ Although largely expressed by activated APCs, the costimulatory function of CD83 seems to inhibit T cell activation, and soluble CD83 inhibits T cell activation by binding to the TLR4/MD-2 complex on monocytes.^[24,25]^

B7-H2 (also known as ICOSL or CD275) is expressed on both professional and nonprofessional APC.^[26–28]^ Activation through toll-like receptor (TLRs) or TNF-α receptors can induce the expression of B7-H2.^[29]^ The binding of B7-H2 and its receptor, the inducible costimulator (ICOS), can elicit the PI3K/AKT signaling pathway, which can in turn activate T cells and enhance T cell proliferation. Unlike B7.1/B7.2, B7-H2 mainly induces the production of the anti-inflammatory cytokine IL-10 instead of IL-2 by T cells, suggesting an immune regulatory role for B7-H2-ICOS.^[26,28,30,31]^ Indeed, the B7-H2-ICOS interaction inhibits both Th1 and Th2 functions.^[32,33]^ However, if T cells over-expressed ICOS, the Th2-derived cytokine IL-10 could be enhanced. The features of ICOS expression in T cell subsets indicates the regulatory role of cytokines releasing with different immunological effects.^[34,35]^ Therefore, the binding of B7-H2 and ICOS has been implicated in the upregulation of regulatory T (Treg) cells.^[36,37]^

B7-H3 was first identified as a costimulatory molecule for T cell activation and interferon-gamma (IFN-γ) and GM-CSF production.^[38]^ B7-H3 mRNA is barely detectable in human peripheral blood mononuclear cells but can be found in various normal tissues and tumor cells.^[39–46]^ Human soluble B7-H3 can induce T cell proliferation and effector cytokine production, including IFN-γ and IL-10.^[38]^ Carcinomas expressing B7-H3 can lead to an anti-tumor response by activating CD8 + T cells.^[47,48]^ In addition, several studies have demonstrated that the interaction between B7-H3 and triggering receptors expressed on myeloid cells (TREM)-like transcript 2 (TLT-2, TREML2) can enhance effector CD8^ + ^T cell activation and function.^[49,50]^

As a newly discovered member of the B7 family, B7-H6 is widely expressed in tumor cells and phagocytes, including monocytes and neutrophils.^[51,52]^ B7-H6 acts as a cell surface ligand for the NKp30-activating receptor expressed on natural killer cells. The interaction of B7H6 with NKp30 results in natural killer (NK) cell activation and cytotoxicity. Another costimulatory molecule, B7-H5, is constitutively found in macrophages and could be induced in DCs. The B7-H5/CD28H interaction selectively stimulates human T cell growth and cytokine production via an AKT-dependent signaling cascade.^[53]^

### 2.2. The role of coinhibitory molecules of b7-CD28 family on interaction between T cells and APCs

The coinhibitory molecules in the B7-CD28 family are also important targets of immune regulation during the adaptive response, such as cytotoxic T lymphocyte antigen-4 (CTLA-4), programmed death-1/programmed death-1 or 2 ligand (PD-1/PD-L1 or 2), B7-H4, B, and T lymphocyte attenuator (BTLA), among others.

CTLA-4 is also a receptor for B7.1/B7.2 and is mainly expressed by Treg cells.^[20,54]^ The administration of a functional blocking monoclonal antibody to CTLA-4 can increase T-cell proliferation both in vitro and in vivo.^[55]^ It has been shown that CTLA-4 exhibits a higher affinity for B7.1/B7.2 than CD28, which can prevent CD28 binding with B7.1/B7.2. Once CTLA-4 engages its ligands, inhibitory intracellular signaling cascades are initiated involving the immunoreceptor tyrosine-based inhibitory motif (ITIM) of CTLA-4 and SHP-2, PP2A, and/or Cbl-b.^[55,56]^ Although there are a number of proposed mechanisms of CTLA-4 coinhibition, the exact mechanism of CTLA-4 coinhibition is not fully understood.

The programmed death-1/programmed death-1 ligand (PD-1/PD-L) interaction was first identified to induce programmed cell death.^[57,58]^ The immunosuppressive function of PD-1 depends on its ITIM and immunoreceptor tyrosine-based switch motif in the cytoplasmic domain.^[59]^ PD-L1 (B7-H1, in peripheral tissues) and PD-L2 (B7-DC, in secondary lymphoid organs) act as ligands for PD-1 during antigen priming and T-cell activation.^[60]^ In addition to being expressed in activated T and B lymphocytes, PD-1 was also found to be inducible in APCs, including activated macrophages, DCs, and monocytes. PD-1 expression on APCs was shown to suppress both adaptive and innate immune responses by inhibiting inflammatory cytokines, such as IL-12 and TNF-α, while promoting the production of the immunosuppressive cytokine IL-10.^[61,62]^ In contrast, the PD-1/PD-L pathway can prevent immunopathology due to lymphocyte overactivation.^[63]^ In addition to the PD-1/PD-L pathway, B7-H3, which binds to TLT-2 for costimulatory functions, may have another role in inhibitory functions, especially in the tumor microenvironment. In glioma studies, tumor cell-bound 4IgB7-H3 molecules suppressed NK cell-mediated tumor cell lysis by interacting with an unknown inhibitory receptor.^[64]^ Recent research has shown that tumor-associated macrophage-related B7-H3 has a strong inhibitory effect on T-cells and influences the outcome of T-cell immune responses.^[65]^ Furthermore, B7-H3 upregulate interleukin-10 (IL-10) secretion and downregulates IL-12 to promote tumor evasion and progression.^[66]^

B7-H4 was identified in 2003 as a new coinhibitory molecule in the B7 family.^[67]^ B7-H4, expressed on the surface of APCs, can inhibit both CD4 + and CD8 + T-cell proliferation, cytokine production, and the generation of effector CTLs.^[68,69]^ B7-H4 can also act as an important negative regulator of innate immunity through growth inhibition of neutrophils.^[70]^ The B7-H4 receptor has not been explicitly revealed, although BTLA was proposed to be the feasible receptor of B7-H4 but was overturned by further research.^[69,71,72]^ Similar to PD-1 and CTLA-4, BTLA is an ITIM-containing inhibitory molecule.^[73,74]^ BTLA can bind to the costimulatory molecules HVEM, CD160, and LIGHT, so CD160/BTLA-HVEM-LIGHT signaling can induce bidirectional functions.^[75,76]^ BTLA-mediated immune inhibition is related to decreased IL-2 production and CD4^ + ^T cell proliferation, but is independent of Treg cell activation.^[73,77]^ B7-H7 also called PD-1H or VISTA, another latest ligand expressed on the surface of monocytes and tumor cells, can inhibit CD4 + and CD8 + T cell function by suppressing proliferation of T cells and production of T cell cytokines.^[78–80]^ However, a study revealed that B7-H7 could also show the costimulatory role by reducing cytokines production including IL-5, IL-10, and IL-13.^[81]^ Regrettably, there have been no relevant studies about the effect of B7-H7 on atherosclerosis up to the beginning of 2022.

## 3. Implications of immune responses in atherosclerosis

The development of atherosclerosis has been described in previous studies.^[82,83]^ Atherosclerosis was always distributed and formed at the incurvation and branching points of the arteries. Endothelial activation leads to lipoprotein permeability, extracellular matrix protein accumulation, and circulating monocyte recruitment. Circulating monocytes differentiate into macrophages and DCs in atherosclerotic plaques.^[84]^

The interaction of T cells and APCs, such as macrophages and DCs, is an important process in activating the immune response and in the development of atherosclerosis. Macrophages and DCs accumulate and bind to T cells in the arterial adventitia and intimal lesions. The maturation of DCs, which is induced by low-density lipoprotein (LDL) in the arterial intima, can overexpress costimulatory molecules and uptake/present antigen proteins. DCs carry the antigen to the lymph node and bind with naïve T cells via antigen peptide–MHC complexes, and then a combination of costimulatory molecules leads to the proliferation, differentiation, and activation of effector T cells. These effector T cells migrate to atherosclerotic lesions, and DCs and macrophages in this region can present the same antigen to activate T cells to promote arterial disorders by secreting inflammatory factors. In this process, costimulatory molecules play important roles in enhancing all T cell subset responses; therefore, costimulatory molecules may be dependent factors in vascular inflammatory diseases, such as atherosclerosis.

Macrophage emigration has been shown to occur in early atherosclerotic plaques, which are activated by cholesterol-mediated oxidation of LDL; LDL is abundant in atherosclerotic plaques.^[85]^ The cholesterol loading of macrophages can overexpress chemokines associated with atherosclerosis, such as netrin 1 and semaphorin 3E.^[86–88]^ In the arterial wall, local T-cell activation and the secretion of proinflammatory cytokines can maintain chronic inflammation and induce macrophage foam cell formation to promote atherosclerosis.^[89,90]^ Macrophage polarization induced by cholesterol is also related to the progression of atherosclerosis. The M1 polarization phenotype of macrophages has been proven to exist in the early stage of atherosclerosis plaque progression, according to the secretion of inflammatory cytokines and chemokines. The M2 phenotype appears to be dominant in the regression of atherosclerosis.

In recent years, the risk factors for oxidized LDL in atherosclerosis have not been fully confirmed whether it is taken up by DCs. One study reported that DCs can take up oxidized LDL by scavenger receptors to mature and release proinflammatory cytokines, such as IL-12.^[91,92]^ Several studies have shown that the accumulation of DCs or CD11c^ + ^monocytes demonstrates the potential impact and initiation of atherosclerosis.^[93–95]^ DCs act as APCs that do not express the costimulatory molecule B7.1/B7.2, reducing atherosclerosis.^[96]^ To date, the mechanisms of action of DCs have not been well defined. However, the function of DCs in atherosclerosis suggests a complex network that controls arterial immune responses.

According to studies of immune factors in atherosclerosis, we can deduce that inflammation and immune responses of T cells play important roles in atherosclerosis. Of course, all the elements in the T cell response may have an effect on atherosclerosis, including T cells, APCs (such as macrophages and DCs), cytokines, and membrane molecules (including costimulatory/coinhibitory and adhesion molecules). As indispensable secondary signals for the activation of T cell response, costimulatory and coinhibitory molecules seem to be potential regulators of atherosclerosis. B7-CD28 superfamily members play the most important roles as costimulatory and coinhibitory molecules; therefore, research on atherosclerosis in these superfamily members has attracted more attention to mechanisms, homeostasis, and atherogenesis.

## 4. B7-CD28 family and atherosclerosis

As an essential process of T cell activation, the integration of costimulatory signals is likely to be a potential target to regulate T cells, currently including immune-related diseases. Atherosclerosis, which belongs to inflammation, is associated with costimulatory or coinhibitory molecules by functional differences given below.

### 4.1. Costimulatory pathway of b7-CD28 family in atherosclerosis

Early studies have shown that B7.1 and B7.2 can be detected in atherosclerotic lesions in both human and mouse model.^[97–100]^ The depletion of B7.1/B7.2 in Ldlr−/− mice decreased IFN-γ production by antigen-specific CD4 + effector T cells and was associated with the reduction of atherosclerotic lesion development.^[96]^ In atherosclerosis, the B7-CD28 costimulatory pathway could influence the functions of proinflammatory effector T cells and Treg suppression depending on the B7.1/2-CD28 axis. However, the confusing results showed that Ldlr−/− mice transferred with B7.1/2−/− bone marrow developed atherosclerosis more easily due to T cell priming relying on less costimulation in bone marrow transplantation.^[101]^ In a recent study, an inhibitory effect on LPS-induced inflammation in atherosclerotic lesions was observed following administration of a specific B7-1 inhibitor. This finding of possible B7-1-dependent negative feedback on APCs may provide a potential therapeutic target for treating atherosclerosis.^[102]^ In a shear stress-induced atherosclerosis mouse model, similar to humans, plaque formation presented macrophage-rich and high levels of B7.1 by positron emission tomography radiotracers.^[103]^ For CD 83, another costimulatory member of the CD28 family, the overexpression of CD83 on DCs in human atherosclerotic lesions was significantly increased in unstable plaques compared to stable plaques, suggesting another potential therapeutic target for atherosclerosis.^[104]^

In atherosclerotic plaques in human and mouse models, ICOS(CD278) and ICOSL(CD275) are abundantly expressed in both adaptive and innate immune cells, such as T cells, macrophages, and DCs.^[97,105–107]^ In contrast to B7.1/B7.2-CD28, the ICOS-ICOSL pathway generally shows an antiatherogenic effect in the formation of atherosclerotic lesions.^[105]^ This is probably because of its dual role in CD4^ + ^Foxp3^ + ^Treg development and immune modulation.^[36,108,109]^ ICOS^−/−^ CD4^ + ^T cells secrete more IFN-γ and TNF-α and less anti-inflammatory cytokines such as TGF-beta, which is associated with increased atherosclerotic lesions in a mouse model of atherosclerosis.^[110]^ In addition, controlling the T follicular helper-germinal center B-cell axis by blocking the CD8^ + ^Treg ICOS-ICOSL pathway could reduce the development of atherosclerosis,^[111]^ and ICOS has an antiatherosclerotic effect by inhibiting aortic smooth muscle cell phagocytosis and proliferation to delay plaque formation in apoE^−/−^ mouse models.^[112]^ Furthermore, in a mouse atherosclerosis model, the expression of ICOS in the spleen was decreased, and promoting ICOS activity could provide a useful strategy for the prevention of atherosclerosis.

For other new costimulatory molecules of B7-CD28 family, there are also relevant members associated with atherosclerosis. B7-H6 in atherosclerotic carotid plaques is abundantly expressed in an HLA-DR + CD11c + myeloid cell population resident.^[113]^ The myeloid differentiation-2(MD-2), which acts as a ligand complex with TLR4, binds to CD83. MD-2 in mouse atherosclerotic lesions can carry cholesterol to activate TLR4 signaling and regulate chronic inflammation in atherosclerosis.^[114–116]^ Inhibition of MD-2 could prevent TNF-α and IL-6 production and reduce nuclear localization of the NF-κB p65 subunit in macrophages to improve atherosclerosis lesions.^[117]^ To date, other known ligand-receptor costimulatory molecules, which played functional roles in cardiovascular atherosclerotic disorders, have been reported, including B7-H3/TLT-2, B7-H5/CD28H, and B7-H6/NKp30.

### 4.2. Coinhibitory pathway of b7-CD28 family in atherosclerosis

Inflammatory responses to hypercholesterolemia can activate APCs expressing costimulatory molecules, which may be regulated by the APCs population.^[118]^ The B7-CD28 pathway of T cells plays a vital role in accelerating the development of atherosclerosis and is strongly negatively regulated by the negative regulator, CTLA-4. The application of CTLA-4 blocking antibodies resulted in an increase in atherosclerotic burden in a hypercholesterolemic mouse model.^[119]^ According to gene analysis, CTLA-4 is part of the IDO-related gene pathway for T cell suppression in human atherosclerotic plaques.^[107]^ Similar to the B7.1/2^*−/−*^Ldlr^*−/−*^ bone marrow chimeras, CD28^*−/−*^Ldlr^*−/−*^ chimeras developed more atherosclerosis, most likely because of the lack of Tregs, which express CTLA-4.^[54]^ In apoE^*−/−*^ mice, CTLA-4-Ig fusion protein, which could reduce T cell activation, resulted in limited neointima formation and reduced homocysteine-accelerated atherosclerosis.^[120]^ Overexpression of CTLA-4 in transgenic mice prevents atherosclerosis.^[121]^ In clinical immunity, the administration of recombinant CTLA-4 has already been used to treat rheumatoid arthritis and transplant rejection, and even to prevent experimental hypertension.^[122]^ However, no clinical data are available regarding the role of CTLA-4 in atherosclerosis.

The expression of PD-ligands in APCs and endothelial cells showed that T cell responses in atherosclerotic lesions may be regulated by the PD/PD-L pathway.^[123,124]^ In PD-L1^−/−^PD-L2^−/−^Ldlr^−/−^ models, atherosclerosis is increased by an extensive immune response of more activated T cells and high levels of serum TNF-α without PD-1 ligands.^[118]^ Chronic exposure to atherosclerosis-related antigens may lead to PD-1 overexpression and CD8 + T cells inhibition. Targeting PD-1 to enhance antiviral immunity may increase cardiovascular risks by activating proatherogenic T cell responses, including atherosclerosis.^[125,126]^ The coexpression of PD-1 and Tim-3 on CD8 + T cells is upregulated in human atherosclerosis, and this T cell subset from the lesional arterial blood produces more anti-atherogenic cytokines. Therefore, promoting PD-1 and Tim-3 pathways may represent novel therapeutic strategies to inhibit atherosclerotic lesion development.^[127]^ In the arterial walls, PD-1 + T cells secrete IFN-γ, IL-17, and IL-21 to drive inflammation-associated angiogenesis and intimal hyperplasia. In the atherosclerotic plaque, PD-L1hi macrophages render patients with coronary artery disease immunocompromised, and the PD-1/PD-L1 immunoinhibitory signal affects vascular inflammation in the atherosclerotic plaque.^[128,129]^ PD-L1 is expressed on microvascular endothelial cells and APCs, such as DCs and macrophages, in the neointima of atherosclerotic lesions.^[119,130]^ It should be illustrated that PD-L1 may downregulate immune responses directly through limited APC-dependent T cell activation in the atherosclerotic tissue.^[131,132]^ For Treg cells activation in atherosclerosis, coinhibitory molecules, such as PD-1/PD-Ligand and CTLA-4, may act as the regulators in atherosclerosis inflammation by cell-to-cell contact and cytokines secretion.^[118,133–135]^

In recent years, there have been few studies on the role of B7-H4 and BTLA in atherosclerosis. But for the combination members of BTLA, HVEM, and LIGHT, as the subjects, had been studied on inflammation atherosclerosis.^[136,137]^ Stimulation of the BTLA pathway in Ldlr^*−/−*^ mice reduced initial atherosclerotic lesion development and increased collagen, possibly by shifting CD4^ + ^T cells towards Treg cells.^[138]^ In the near future, research on these coinhibitory molecules will play an important role in the formation and development of atherosclerosis.

## 5. Outlook: potential targets of b7-CD28 family in atherosclerosis

Atherosclerosis is currently viewed as a chronic inflammatory cardiovascular disease. Increasing evidence has suggested that targeting immune components (T cells,^[139,140]^ macrophages,^[86,141–144]^ and DCs^[144,145]^) of atherosclerosis may be a potential therapeutic strategy for treating diseases. Costimulatory and coinhibitory molecules serve as important modulators of the T cell response. These molecules regulate the occurrence and progression of atherosclerosis. Among them, the B7-CD28 family members also play important roles in the normal immune surveillance of infections or tumors. Therefore, to avoid potential complications of the treatment, future studies should focus on plaque-specific drug delivery of costimulatory signal inhibitors.

Atherosclerosis is considered to be a chronic inflammatory disease with T cell activation, which may be beneficial for atherosclerotic progression. Therefore, costimulation blocking or coinhibitory activation strategies may be effective in atherosclerosis, and preclinical evidence supports costimulation-blocking therapies. The B7-CD28 pathway is considered a primary target in immune-related diseases, including vascular pathology. The combination of B7 and CD28 molecules was the strongest second signal for T cell response. Therefore, according to the clinical data, circulating CD28(null) T cells may be strongly associated with the recurrence of cardiovascular diseases and play a key pathogenetic role. So targeting B7 or CD28 of T cells may be a potentially possible approach for vascular disorders, such play as by regulating CD28(null) T cells.^[146,147]^ Research on atherosclerosis in human arteries has shown that the expression levels of costimulatory molecules CD80 and CD86 in atherosclerotic plaques have significantly increased, so CD80 and CD86 expressed by activated APCs are promising imaging targets in atherosclerosis.^[102,148]^ In addition, different costimulatory pathways in atherosclerosis may influence disease stage. For example, naïve CD4^ + ^T cells may contribute to the development of early lesions, which are regulated by CD28 signaling. However, effector and memory T cells within lesions may be influenced by ICOS and PD-1 pathways. Therefore, costimulatory blockade can impair both effector and regulatory T cell differentiation and function; thus, the modulation of these molecules may be difficult.

If T cells received antigen receptor signals without costimulatory signals, they could not lead to the specific activation that induced immunological tolerance. In the following decade, studies on blood vessel diseases and effective strategies for tolerance induction based on costimulatory blockade appeared to be attainable.^[149]^ As a chronic disease, atherosclerosis has raised questions about the practicality of immune therapy in the costimulatory and coinhibitory pathways of the B7-CD28 family. According to immunology theory, the ideal immunological approach for atherosclerosis is to induce long-lasting specific T cell tolerance to atherosclerosis-specific antigens. Therefore, the application of a costimulatory blocking or a coinhibitory agonist agent of the B7-CD28 family for specific antigen tolerance induction is theoretically feasible.

Previous research has identified costimulatory or coinhibitory molecules as important modulators of the immune response. Recent immune-related bioinformatics analysis of atherosclerosis has shown that the progression of atherosclerosis may be linked to multiple immuno-checkpoint molecular genes, including members of the B7-CD28 superfamily. These genes may play important roles in regulating immune cells, such as monocytes/macrophages, T cells, and B cell subsets, and may serve as potential therapeutic targets for atherosclerosis.^[150]^ As the most important and strongest functional family in costimulatory and coinhibitory pathways, the B7-CD28 family has, not surprisingly, been the potential genetic and pharmacological manipulation target in atherosclerosis. These molecules, located on the surface of both T cells and APCs, including macrophages and DCs, play significant roles in the process of atherosclerosis. Clinical application of monoclonal antibodies to PD1, PD-L1, PD-L2, and CTLA-4 confirmed the successful manipulation of the B7-CD28 coinhibitory pathways. Although the exact mechanisms and molecular understanding of the B7-CD28 family in atherosclerosis are not definite, these pathways will remain critical, and accelerated research programs should come into focus on this chronic vessel inflammatory disease.

## Author contributions

YM, TS, and LZ were responsible for the collection and classification of literature. YM and TS were responsible for writing the manuscript. FZ and LC are responsible for the establishment of the content of the review, embellishment of manuscripts, and establishment of highlights.

**Data curation:** Mao Yang.

**Funding acquisition:** Mao Yang.

**Investigation:** Simeng Tian, Zhoujun Lin, Zhenkun Fu.

**Supervision:** Zhenkun Fu, Chenggang Li.

**Writing – original draft:** Simeng Tian, Mao Yang.

**Writing – review and editing:** Chenggang Li, Zhenkun Fu.
